# Clinical Diagnostic Bacteriology, Including Serum Diagnosis and Cyto-Diagnosis

**Published:** 1904-12

**Authors:** 


**Clinical Diagnostic Bacteriology,** including Serum Diagnosis and Cyto-diagnosis. By Alfred C. Coles, M.D., D.Sc. Pp. viii., 233. London: J. & A. Churchill. 1904.

The author describes this work as "a small book with an ambitious title," and explains that it is intended as a handbook for general practitioners who, without special laboratory training, are anxious to avail themselves of modern laboratory methods in the diagnosis of disease. Only a practitioner so placed, who has made a practical trial of this book, could say how far the author has attained his principal aim, but it certainly appears to us that for those who have *some* previous acquaintance with bacteriological technique, this volume will be found useful and convenient. The various technical methods are simply and lucidly described, and the limits of their use in diagnosis and prognosis are indicated—an important point which is apt to be overlooked, especially by the inexperienced investigator.

More than one-third of the book is devoted to the author's original researches on the differentiation by staining reactions of the various acid-fast bacilli, which are likely to be confused with b. tuberculosis. His experiments and results are extremely interesting, but will of course require confirmation by experienced investigators; in fact, this portion of the book, which is the most valuable in a general sense, seems hardly in consonance with the scope of the work as indicated in the preface.

There are two very good coloured plates representing stained microscope specimens of various bacteria and other parasites, but both in the coloured plates and in the text a little more detailed description of the various forms of the diphtheria bacillus might have been given with advantage. The "barred" forms are not very distinctly described in the text, and in the plates the fine, long bacilli with two or more granules stained by Loeffler's blue are not figured. An illustration of Hofmann's bacillus (which is spelt "Hoffmann") would be a valuable addition.

The account of the diphtheria organisms illustrates again the confusion which is produced by the use of that unfortunate prefix "pseudo." Dr. Coles restricts it to the Hofmann-Wellenhof organisms; many authors include the Xerosis bacillus under this term, and by others it is loosely used to describe any non-virulent or slightly virulent organism resembling the true Klebs-Loeffler bacilli. In some of the quotations given from other writers it is not clear whether the term is used in the more restricted or the wider sense.

The chapters on Serum Diagnosis and Cyto-diagnosis form a useful introduction to these subjects. The details of the microscopical preparations involved might perhaps be a little fuller, but references are given to easily-accessible papers which will sufficiently supplement the text. The work on the whole is of an eminently practical character, and in this lies its main value to the student of clinical bacteriology, to whom it may safely be recommended as a useful technical guide.

